# Facile In Situ Synthesis of Self-Supporting Cu Nanoparticles/Nickel Foam Electrode for Sensitive Non-Enzymatic Electrochemical Glucose Sensing in Beverages

**DOI:** 10.3390/foods15111993

**Published:** 2026-06-03

**Authors:** Yanlin Wu, Xintian Ma, Yiyue Ma, Jianlong Wang

**Affiliations:** College of Food Science and Engineering, Northwest A&F University, Yangling 712100, China; wyl2005@nwafu.edu.cn (Y.W.); 1744625973@nwafu.edu.cn (X.M.)

**Keywords:** non-enzymatic glucose sensor, glucose detection, beverage, Cu nanoparticles, Ni foam, electrochemical sensor

## Abstract

Accurate quantification of glucose is vital for quality control in the food industry. While earth-abundant Cu has emerged as a promising candidate for non-enzymatic electrochemical sensing, conventional electrode fabrication relying on powder coating with polymeric binders inevitably buries active catalytic sites and impedes both electron transfer and mass transport. In this study, a binder-free, self-supporting Cu nanoparticles/Ni foam (Cu NPs/NF) electrode was developed via a facile one-step hydrothermal method. Benefitting from the enhanced charge-transfer efficiency and a substantially enlarged electrochemical active surface area, the Cu NPs/NF-based electrochemical glucose sensor exhibited a wide linear detection range (0.25–3310.52 μM), a high sensitivity of 7000 μA mM^−1^ cm^−2^, a low detection limit of 0.32 μM, and a rapid response time of 3 s. Furthermore, the developed Cu NPs/NF electrode displayed favorable reproducibility, storage stability, and high selectivity against common interferents present in food matrices, demonstrating its reliability for practical applications. The feasibility of the proposed sensor was successfully validated in real beverage samples. Given the simplicity of the one-step hydrothermal synthesis and the portability afforded by the self-supporting electrode architecture, this Cu NPs/NF electrode emerges as a highly attractive candidate for commercial glucose sensors. Beyond glucose, the design strategy can be readily extended to the detection of other electroactive food-quality markers, enabling the broader applicability of this electrode platform in comprehensive food analysis.

## 1. Introduction

Glucose is an essential monosaccharide and an indispensable energy source for cellular metabolism in living organisms [1]. Beyond its critical role in clinical diagnostics for diabetes management, the accurate quantification of glucose holds significant economic and regulatory importance in the food industry. It is crucial for assessing caloric content, sweetness index, and fermentation efficiency in products such as beverages, dairy, honey, and baked goods [2,3]. Consequently, various analytical techniques have been developed for glucose detection, including high-performance liquid chromatography (HPLC), colorimetry, fluorescence spectroscopy, surface-enhanced Raman scattering (SERS), and surface plasmon resonance (SPR)-based sensors [4,5,6,7,8,9]. While these methods offer high precision, their practical application in food processing environments is often constrained by bulky instrumentation, complex sample pretreatment, high operational costs, and the requirement for skilled technicians, thereby hindering real-time and on-site glucose monitoring. Electrochemical sensors have emerged as a promising alternative owing to their rapid response, operational simplicity, low cost, and excellent compatibility with turbid or colored food matrices [10,11].

Electrochemical glucose sensors are broadly classified into enzymatic and non-enzymatic systems. Although enzymatic sensors based on glucose oxidase (GOx) possess exceptional selectivity, the complexity of the enzyme immobilization process and the intrinsic instability of enzymes, leading to issues such as inactivation at high temperatures, pH sensitivity, and limited shelf life, limit their practical application in food industry [12]. In contrast, non-enzymatic electrochemical glucose sensors, which rely on the direct electrocatalytic oxidation of glucose at the electrode surface, have emerged as compelling alternatives owing to their superior stability, lower fabrication cost, and simplified operational procedures [13,14].

Traditionally, noble metals and their alloys (e.g., Pt, Au, Pd) have been extensively explored as electrode materials for direct glucose oxidation due to their superior catalytic activity [15,16]. However, the practical deployment of noble metal-based sensors is severely constrained by high material costs. Consequently, earth-abundant transition metal-based nanomaterials, including oxides, hydroxides, and metallic nanoparticles, have been extensively investigated as promising electrode materials. Among these, copper-based nanostructures have attracted particular attention due to the favorable Cu(II)/Cu(III) redox couple, high natural reserves, and excellent electrocatalytic activity toward glucose oxidation in alkaline media [17,18]. However, conventional electrode fabrication methods for copper-based materials typically involve the synthesis of powders followed by drop-casting onto planar current collectors with the aid of polymeric binders. This approach inevitably buries a substantial fraction of the catalytic active sites beneath an insulating polymer matrix and impedes interfacial electron transfer and mass transport, thereby limiting the achievable sensitivity and dynamic response [19,20].

To overcome these limitations, significant efforts have been directed toward the development of binder-free, self-supporting electrode architectures wherein the active material is directly grown on three-dimensional conductive substrates such as nickel foam (NF), carbon cloth, or metal nanowire arrays [21,22]. Such integrated configurations not only eliminate the need for insulating binders but also leverage the macroporous conductive framework to facilitate rapid electron collection and unimpeded electrolyte diffusion. Despite these advances, many reported synthetic routes need prolonged hydrothermal treatments, multi-step procedures, or the use of sacrificial templates, which compromise scalability and cost-effectiveness. Therefore, a facile, rapid, and scalable synthesis strategy for constructing high-performance copper-based self-supporting electrodes remains highly desirable.

**Scheme 1:**
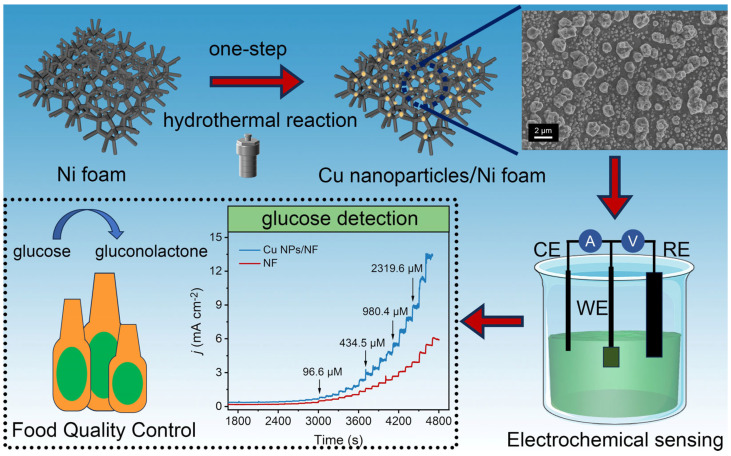
Illustration of the fabrication of the Cu NPs/NF electrode and its application for glucose detection.

Herein, we report a strategy that fundamentally addresses these structural and electronic limitations through the rational design of a binder-free, self-supporting Cu nanoparticles/nickel foam (Cu NPs/NF) integrated electrode fabricated via a facile one-step hydrothermal method (Scheme 1). This approach offers distinct advantages compared to conventional ex situ powder coating techniques. The direct in situ nucleation and growth of Cu NPs on the NF skeleton ensures intimate mechanical adhesion, which minimizes charge-transfer resistance without the insulating barrier of a polymeric binder. In addition, the robust three-dimensional (3D) macroporous architecture of the NF substrate serves as a continuous conductive highway, enabling rapid electron collection from the active Cu NPs while simultaneously facilitating the unimpeded diffusion of glucose molecules and electrolyte ions. Consequently, the as-prepared Cu NPs/NF electrode demonstrates significantly enhanced electrocatalytic performance toward glucose oxidation. Electrochemical characterization confirmed that the Cu NPs/NF-based sensor serves as an efficient platform for glucose detection, exhibiting a wide linear range, a high sensitivity, a low detection limit, and a fast response time, as well as excellent reproducibility, outstanding selectivity, and satisfactory storage stability. Importantly, the Cu NPs/NF electrode also demonstrated exceptional reliability in beverage sample analyses, confirming its potential for practical food quality monitoring. This work not only presents a high-performance sensing platform suitable for food quality assurance but also underscores the pivotal role of 3D hierarchical electrode architecture in maximizing the electrochemical utilization of earth-abundant transition metal catalysts.

## 2. Experimental

### 2.1. Materials

Glucose, NaOH, CuSO_4_, KCl, Na_2_SO_4_, Zn(NO_3_)_2_, MgCl_2_, and CaCl_2_ were purchased from Guanghua Technology Co., Ltd. K_3_[Fe(CN)_6_] and K_4_[Fe(CN)_6_] were purchased from Sigma-Aldrich Co., Ltd. (Shanghai, China). A commercial glucose assay kit based on the glucose oxidase–peroxidase (GOD-POD) method was purchased from Beyotime Biotech Inc. (Shanghai, China). The beverage sample (a commercial glucose oral rehydration solution) was purchased from a local supermarket. Analytical grade chemicals were used directly as supplied. All aqueous solutions were prepared with deionized (DI) water.

### 2.2. Preparation of Cu NPs/NF Electrode

The self-supporting Cu NPs/NF electrode was fabricated via a facile one-step hydrothermal method. In a typical synthesis procedure, a piece of nickel foam (NF) was cut into 2 cm × 3 cm. Then it was cleaned by acid etching in 3 M HCl solution for 4 min and ultrasonication in deionized (DI) water for 5 min to remove the surface oxide layer. The surface moisture of the cleaned NF substrate was then dried by filter paper. Subsequently, 0.05 g of CuSO_4_ 5H_2_O were dissolved in 40 mL of DI water under continuous magnetic stirring to form a homogeneous light-blue solution. This solution was transferred into a 50 mL Teflon-lined stainless-steel autoclave, into which the pretreated NF substrate was vertically immersed. The autoclave was sealed and maintained at 150 °C for 30 min in an electric oven to facilitate the in situ nucleation and growth of Cu nanoparticles directly onto the NF skeleton. Upon natural cooling to ambient temperature, the resulting Cu NPs/NF electrode was carefully rinsed with DI water to eliminate loosely attached species, and finally dried at 80 °C for 10 h. The as-prepared electrode was employed directly as the working electrode without the addition of any polymeric binders or conductive additives.

### 2.3. Characterizations

The crystal structures of the catalysts were characterized by X-ray diffraction (XRD, Bruker D8 ADVANCED, Karlsruhe, Germany) using Cu Kα radiation (40 kV, 40 mA). Morphological and structural features of the synthesized materials were examined using scanning electron microscopy (SEM, Hitachi S-4800, Yokohama, Japan) equipped with energy-dispersive X-ray spectroscopy (EDS) for elemental mapping. The surface chemical states were analyzed by X-ray photoelectron spectroscopy (XPS, ESCALAB MK II, VG Scientific, London, UK) with an Mg Kα X-ray source.

### 2.4. Electrochemical Measurement

Electrochemical measurements were carried out by a CHI660E electrochemical workstation (Chenhua, Shanghai, China) using a standard three-electrode system with a platinum wire as the counter electrode, a HgO electrode as the reference electrode, and the synthesized Cu NPs/NF as the working electrode. A 0.1 M NaOH solution served as the electrolyte, with the exposed geometric area of the working electrode fixed at 0.5 × 0.5 cm^2^. Cyclic voltammetry (CV) was conducted within a potential window of 0–1.0 V at a scan rate of 50 mV s^−1^. Electrochemical impedance spectroscopy (EIS) curves were measured at the open-circuit potential over a frequency range of 0.01 Hz to 1 MHz. Amperometric i–t curves were recorded under constant applied potential and continuous stirring, with successive glucose additions introduced at 100 s intervals. All reported electrochemical data represent the mean ± standard deviation obtained from at least three independently prepared electrodes, with each electrode measured in triplicate.

## 3. Result and Discussion

### 3.1. Materials Characterization

**Figure 1:**
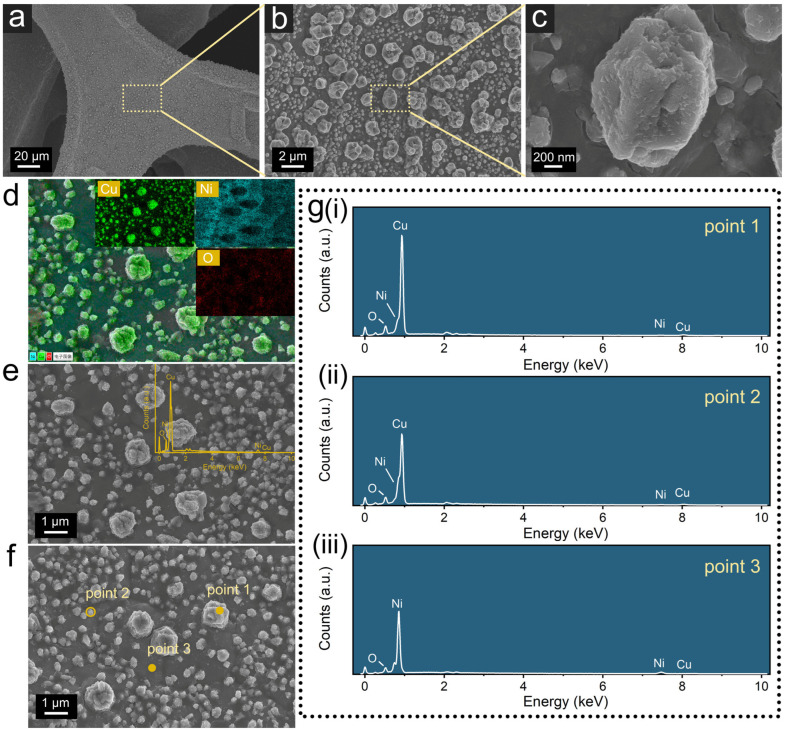
(**a**–**c**) SEM images of Cu NPs/NF at (**a**) low magnification, (**b**) medium magnification, and (**c**) high magnification. (**d**) EDX elemental mapping images of Cu NPs/NF. (**e**) EDX spectrum acquired from the entire field of Cu NPs/NF. (**f**,**g**) EDX point-scan spectra collected from three representative nanoparticle sites (marked as point 1, point 2, and point 3).

The self-supporting Cu NPs/NF electrode was fabricated via a facile one-step hydrothermal method. The surface morphology of the as-prepared electrode was characterized by SEM. As illustrated in Figure 1a,b, the hydrothermal treatment led to the in situ growth of Cu nanoparticles, which were uniformly distributed onto the three-dimensional macroporous skeleton of the NF substrate. Figure 1c further presents a high-magnification SEM image of individual Cu nanoparticles, and the particle size distribution of the Cu NPs was also analyzed (Figure S1). The formation of a hierarchical nanostructure on the electrode surface can provide abundant active sites and facilitate interfacial electrochemical reactions. To verify the successful incorporation of Cu species onto the NF surface, EDX elemental mapping and the corresponding elemental distribution maps were performed over a representative region. As shown in Figure 1d,e, the results reveal a continuous Ni signal from the foam backbone overlaid with a uniform and intense Cu signal, confirming the introduction of Cu during the hydrothermal process and its dense and homogeneous distribution throughout the three-dimensional skeleton. In addition, point-scan EDX analyses were carried out at different representative regions of the sample to further elucidate the local elemental composition of individual nanoparticles. As presented in Figure 1e,f, the nanoparticles were predominantly composed of Cu element, further confirming the successful formation of Cu-containing nanostructures on the nickel foam substrate.

**Figure 2:**
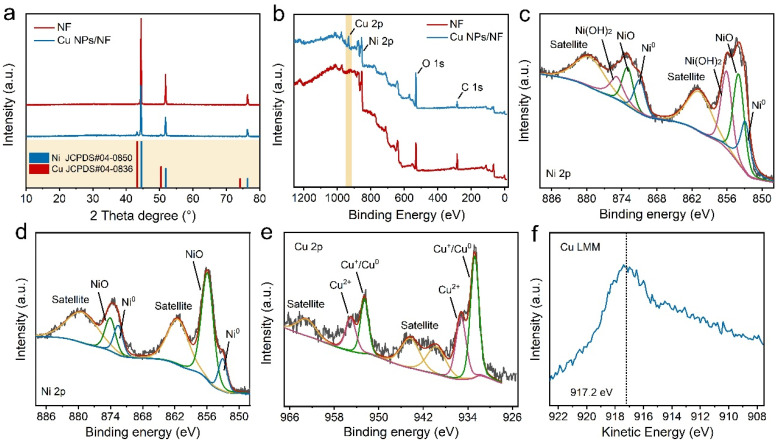
(**a**) XRD patterns of NF and Cu NPs/NF. (**b**) XPS survey spectra of NF and Cu NPs/NF. (**c**) XPS high-resolution Ni 2p spectrum of NF. (**d**) XPS high-resolution Ni 2p spectrum of Cu NPs/NF. (**e**) XPS high-resolution Cu 2p spectrum of Cu NPs/NF. (**f**) Cu LMM Auger spectrum of Cu NPs/NF.

The crystalline phase and surface chemical composition of the as-synthesized Cu NPs/NF electrode were investigated using XRD and XPS. Figure 2a presents the XRD patterns of the bare nickel foam (NF) and the Cu NPs/NF electrode. In addition to the three intense diffraction peaks located at 44.4°, 51.8°, and 76.3° which respectively correspond to the (111), (200), and (220) planes of metallic Ni (JCPDS 04-0850) [23], three additional characteristic reflections emerged at 43.2°, 50.4° and 74.0° for the Cu NPs/NF sample. These peaks are indexed to the (111), (200), and (220) planes of metallic Cu (JCPDS 04-0836) [24], confirming the in situ growth of crystalline Cu on the NF skeleton.

The surface elemental composition and valence states were further elucidated by XPS analysis. The XPS survey spectra in Figure 2b clearly exhibit the characteristic signals of C, O, Ni, and Cu, demonstrating the successful incorporation of Cu species onto the NF surface. The high-resolution Ni 2p spectrum of the pure NF electrode is shown in Figure 2c. The spectrum exhibits two main peaks at 854.4 eV (Ni 2p_3/2_) and 873.1 eV (Ni 2p_1/2_), accompanied by their respective satellite features at higher binding energies (861.1 eV and 879.8 eV). Deconvolution of the high-resolution Ni 2p spectrum reveals the coexistence of three distinct chemical states on the surface of NF. The two peaks located at binding energies of approximately 852.9 eV and 871.0 eV can be assigned to metallic Ni^0^, originating from the nickel foam substrate. The two peaks centered at 854.1 eV and 873.1 eV correspond to NiO, which constitutes the native surface oxide layer. In addition, the two peaks observed at higher binding energies of 856.2 eV and 875.0 eV can be attributed to Ni(OH)_2_, indicating partial hydration of the surface oxide upon exposure to ambient moisture [25]. In contrast, the Ni 2p spectrum of the Cu NPs/NF electrode (Figure 2d) reveals that the characteristic peaks associated with Ni(OH)_2_ are absent, while the signals corresponding to Ni^0^ and NiO are retained, suggesting that surface Ni(OH)_2_ domains may serve as preferential sacrificial sites for the heterogeneous nucleation and growth of Cu NPs.

Figure 2e displays the high-resolution Cu 2p spectrum of the Cu NPs/NF electrode. The two predominant asymmetric peaks correspond to Cu 2p_3/2_ and Cu 2p_1/2_ spin-orbit components, respectively, and are accompanied by intense shake-up satellite peaks. The spectrum can be deconvoluted into Cu^2+^ at binding energies of ~935.2 eV and ~955.0 eV and Cu^+^/Cu^0^ at binding energies of ~932.8 eV and ~952.6 eV [26,27]. To distinguish the Cu^+^ and Cu^0^ with similar binding energies in high-resolution Cu 2p spectrum, the Cu LMM Auger spectrum of Cu NPs/NF was measured (Figure 2f). There is an obvious peak at kinetic energy of 917.2 eV related to metallic Cu^0^, providing conclusive evidence for the formation of the Cu phase [26]. Collectively, the XPS and Auger analyzes reveal that the Cu NPs/NF electrode comprises a mixed-phase copper surface, wherein metallic Cu^0^ coexists with a minor fraction of Cu^2+^ (Figure S2), which can be attributed to the surface oxidation of the Cu^0^ nanoparticles upon exposure to ambient air during sample handling.

The above characterization results collectively demonstrate that Cu NPs were successfully synthesized and were uniformly dispersed across the three-dimensional porous framework of NF, which is highly advantageous for electrochemical sensing applications.

### 3.2. Electrochemical Performance

#### 3.2.1. Electrochemical Characterization of Cu NPs/NF

**Figure 3:**
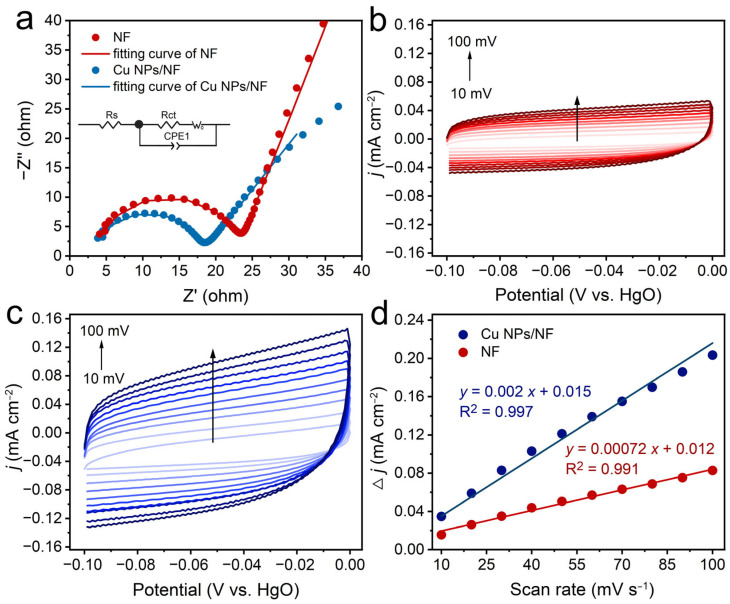
(**a**) Nyquist curves of NF and Cu NPs/NF. (**b**) CV curves of NF in 0.1 M NaOH at different scan rates. (**c**) CV curves of Cu NPs/NF in 0.1 M NaOH at different scan rates. (**d**) Calculation of *C*_dl_ of NF (red line) and Cu NPs/NF (blue line) in 0.1 M NaOH.

To elucidate the interfacial charge-transfer characteristics of the NF and Cu NPs/NF electrodes, EIS measurements were conducted in a 0.1 M KCl solution containing 5 mM [Fe(CN)_6_]^3−/4−^ as the redox probe (Figure 3a). Both of the Nyquist plots of the NF and Cu NPs/NF electrodes exhibit a semicircular region in the high frequency corresponding to the charge-transfer process at the electrode/electrolyte interface, which is followed by a Warburg curve in the low-frequency region. As shown in the inset of Figure 3a, the EIS curves were fitted by a Randles-type equivalent circuit model composed of an uncompensated solution resistance (*R*_s_), a charge-transfer resistance (*R*_ct_), a constant phase element (CPE), and a Warburg impedance (*W*_o_). The fitting results reveal that the *R*_ct_ value of the Cu NPs/NF electrode (~12.64 Ω) was lower than that of the pure NF (~19.18 Ω), indicating the improvement in interfacial electron-transfer efficiency, which may be attributed to the introduction of highly conductive Cu species. In the low-frequency region of the Nyquist plot, the Cu NPs/NF electrode exhibits a steeper Warburg tail than the pure NF electrode, indicating enhanced electrolyte diffusion enabled by the three-dimensional macroporous architecture formed upon Cu NP loading. In addition, the electrochemically active surface area (ECSA) can be estimated by the CV curves at varying scan rates in a strictly non-Faradaic potential window [28]. As exhibited in Figure 3b,c, the CV curves of NF and Cu NPs/NF electrodes were recorded in 0.1 M NaOH solution at scan rates ranging from 10 to 100 mV s^−1^. The capacitive current density at −0.05 V was plotted against the scan rates (Figure 3d), and the slope of the obtained linear relationship corresponds to the specific double-layer capacitance. The Cu NPs/NF electrode exhibits a *C*_dl_ value of 1 mF cm^−2^, which is 2.8-fold higher than that of pure NF. As there is direct proportional correlation between *C*_dl_ and ECSA, the much higher *C*_dl_ value of the Cu NPs/NF electrode indicates a substantially enlarged ECSA following the loading of Cu NPs, which can enhance the sensitivity and current response in glucose sensing.

The above results confirm that the formation of Cu NPs and their uniform dispersion on the NF substrate can facilitate efficient electron transport while simultaneously providing abundant accessible active sites for interaction with the analyte, thereby contributing to the enhanced sensing performance.

#### 3.2.2. Electrocatalytic Activity of Cu NPs/NF

**Figure 4:**
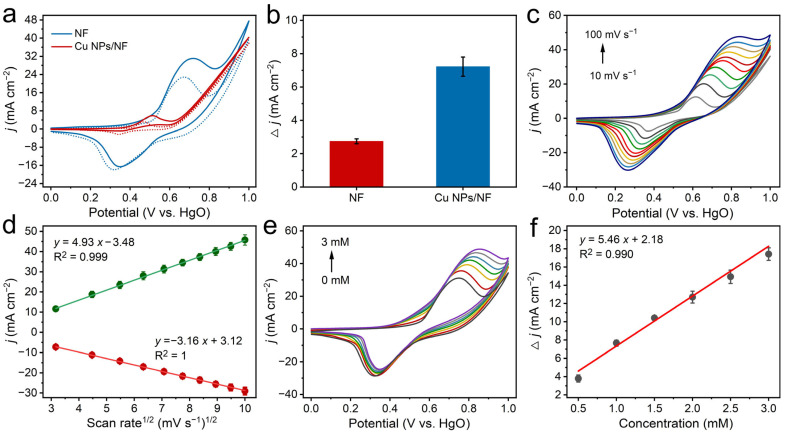
(**a**) CV curves of NF and Cu NPs/NF electrodes recorded in 0.1 M NaOH (dash line) and in 0.1 M NaOH containing 1 mM glucose (solid line) and (**b**) the corresponding column chart of anodic peak current density. (**c**) CV curves of the Cu NPs/NF electrode in 0.1 M NaOH containing 1 mM glucose at scan rates ranging from 10 to 100 mV s^−1^ and (**d**) corresponding linear plots of the anodic peak (green line) and cathodic peak (red line) current densities versus the square root of the scan rate. (**e**) CV curves of the Cu NPs/NF electrode in 0.1 M NaOH containing different concentrations of glucose (0–3 mM) and (**f**) corresponding linear calibration plot of the anodic peak current density versus the concentration of glucose. Data are presented as mean ± SD (*n* = 3 independent electrodes).

Cyclic voltammetry (CV) was employed to evaluate the electrocatalytic activity of the NF and as-prepared Cu NPs/NF electrodes toward glucose oxidation. As shown in Figure 4a, the CV curves were recorded in 0.1 M NaOH solution in the presence of 1.0 mM glucose. In the absence of glucose, a weaker background current was observed for both electrodes. Upon glucose addition, the Cu NPs/NF electrode exhibited a well-defined anodic peak at ~0.717 V and a corresponding cathodic peak at ~0.348 V. Compared with the pure NF electrode, the Cu NPs/NF electrode displayed a significantly enhanced response current density (Figure 4b), indicating its superior electrocatalytic performance toward glucose oxidation, which can be ascribed to the abundant accessible Cu active sites and the highly conductive three-dimensional scaffold. Figure S3 illustrates the mechanism of the electro-oxidation process of glucose on the surface of Cu NPs/NF electrode. The metallic Cu^0^ NPs provides high electrical conductivity and facilitates rapid electron collection, while the surface Cu atoms are partially oxidized to Cu^2+^ species in alkaline medium. Then the surface Cu^2+^ species are subsequently electrochemically oxidized to CuOOH, in which the Cu center attains a highly oxidizing Cu^3+^ state. Meanwhile, glucose molecules undergo deprotonation and isomerize into an enediol form. The enediol then contacts Cu^3+^ species and is oxidized to gluconolactone [18]. During this process, Cu^3+^ is reduced back to Cu^2+^, and the electron released from glucose oxidation is collected through the underlying metallic Cu^0^ core and the three-dimensional NF scaffold, completing the catalytic cycle and generating the anodic current signal. In addition, to investigate the reaction kinetics in glucose oxidation at the interface of Cu NPs/NF, CV measurements were carried out at various scan rates ranging from 10 to 100 mV s^−1^ in 0.1 M NaOH containing 1 mM glucose. Figure 4c illustrates a progressive positive shift of the anodic peak potential with increasing scan rates, indicating finite electron-transfer kinetics characteristic of a quasi-reversible process [29]. As shown in Figure 4d, a linear relationship was established between the anodic peak current and the square root of the scan rates, demonstrating a diffusion-controlled reaction mechanism in the electro-oxidation of glucose at the Cu NPs/NF electrode [30].

The quantitative sensing performance of the Cu NPs/NF electrode was further examined by recording CV curves in 0.1 M NaOH solution containing different concentrations of glucose. As depicted in Figure 4e,f, the anodic peak current density increased linearly within the glucose concentration window of 0–3 mM, following the regression equation *j_pa_* (mA cm^−2^) = 5.46 *C* (mM) + 2.18 (R^2^ = 0.990). These findings underscore the notable electrocatalytic activity of Cu NPs/NF in glucose oxidation and validate its promising potential in electrochemical sensing applications.

#### 3.2.3. Electrochemical Sensing Performance of Cu NPs/NF Electrode

**Figure 5:**
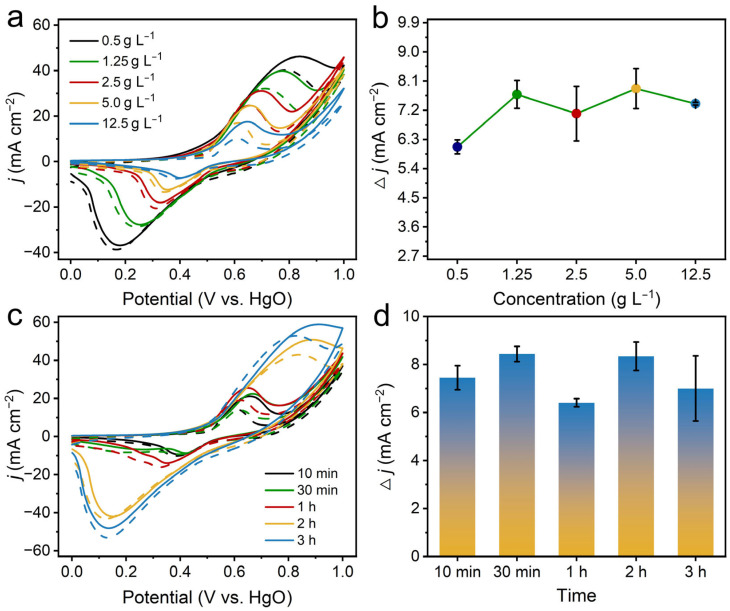
(**a**) CV curves of Cu NPs/NF electrodes prepared with different concentrations of CuSO_4_ (dash line: recorded in 0.1 M NaOH, solid line: recorded in 0.1 M NaOH containing 1 mM glucose) and (**b**) the corresponding line chart of response current density. (**c**) CV curves of Cu NPs/NF electrodes prepared for different lengths of time in hydrothermal reaction (dash line: recorded in 0.1 M NaOH, solid line: recorded in 0.1 M NaOH containing 1 mM glucose) and (**d**) the corresponding column chart of response current density. Data are presented as mean ± SD (*n* = 3 independent electrodes).

To optimize the synthesis parameters of the Cu NPs/NF electrode, a series of electrodes were prepared by varying the CuSO_4_ concentration and the hydrothermal reaction time. Figure 5a displays the CV curves recorded in 0.1 M NaOH solution in the absence and presence of glucose for electrodes synthesized using different CuSO_4_ concentrations. As the CuSO_4_ concentration increases, the background current gradually decreases, and a progressive negative shift in the oxidation peak potential is observed. This negative shift indicates an improved electrocatalytic activity toward glucose oxidation, which can be attributed to the increased formation and loading of Cu NPs onto the NF substrate at higher CuSO_4_ concentrations. The corresponding column chart presented in Figure 5b reveals that the response current density exhibits only marginal variation when the CuSO_4_ concentrations increases to 1.25 g L^−1^. In consideration of the electrocatalytic activity and the response current density, a CuSO_4_ concentration of 5 g L^−1^ was chosen as the optimal condition for the hydrothermal synthesis.

Furthermore, the hydrothermal reaction time was also optimized. As shown in Figure 5c, when the reaction time exceeded 1 h, the background current increased and the oxidation peak potential shifted toward higher values, indicating a decline in electrocatalytic performance toward glucose oxidation. According to the column chart in Figure 5d, the electrodes prepared with reaction times of 30 min and 2 h exhibited relatively higher response current densities. Considering the fabrication efficiency and sensing performance, a hydrothermal reaction time of 30 min was finally adopted as the optimal synthesis condition.

In summary, the optimization study indicated that the best electrochemical performance of Cu NPs/NF electrode was achieved with a CuSO_4_ concentration of 5 g L^−1^ and a reaction time of 30 min. The enhanced electrocatalytic behavior may be attributed to the combination of the highly conductive NF framework and the well-dispersed Cu NPs with high catalytic activity, which collectively promote efficient electron transfer and facilitate the electro-oxidation of glucose.

**Figure 6:**
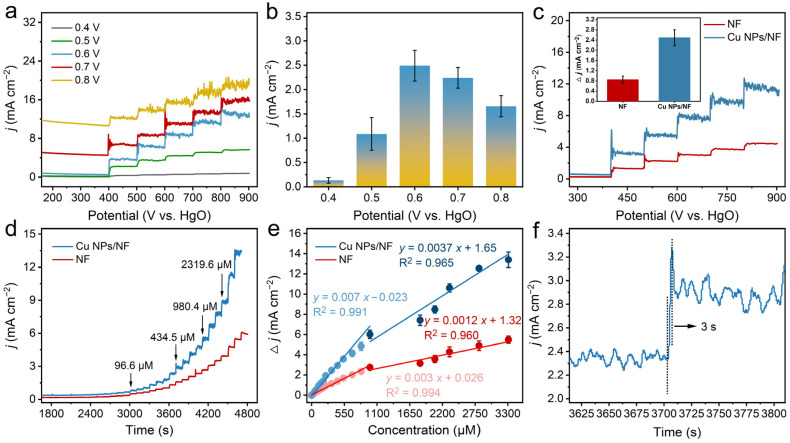
(**a**) Amperometric i–t curves of the Cu NPs/NF electrode recorded in 0.1 M NaOH upon successive additions of glucose at different applied potentials. (**b**) Corresponding column chart of the response current density at each applied potential. (**c**) Amperometric i–t curves of NF and Cu NPs/NF electrodes in 0.1 M NaOH upon addition of 350 μM glucose. Inset: corresponding column chart of the average response current density. (**d**) Amperometric i–t curves of NF and Cu NPs/NF electrodes in 0.1 M NaOH with successive addition of glucose over a wide concentration range. (**e**) Corresponding calibration curves of the response current density versus glucose concentrations. (**f**) Response time of the Cu NPs/NF electrode upon glucose addition in amperometric measurement. Data are presented as mean ± SD (*n* = 3 independent electrodes).

To establish optimal detection parameters, the amperometric performance of the Cu NPs/NF electrode was evaluated at various applied potentials ranging from 0.4 to 0.8 V. As presented in Figure 6a, the i-t curves were recorded in 0.1 M NaOH solution upon successive additions of glucose. The corresponding column chart reveals that the response current increases progressively as the applied potential is raised from 0.4 to 0.6 V (Figure 6b). The applied potential of 0.6 V yields the maximal current response among all potentials examined. When the potential exceeds 0.6 V, the response current declines and is accompanied by an increased background noise. Considering the signal stability, current response, and energy consumption, an applied potential of 0.6 V was identified as the optimal working potential and adopted for all subsequent measurements. To further elucidate the superior sensing performance of the Cu NPs/NF electrode, amperometric measurements using both the bare NF and Cu NPs/NF electrodes were performed in 0.1 M NaOH upon four consecutive additions of 350 μM glucose. As shown in Figure 6c, upon each glucose injection, the Cu NPs/NF electrode exhibited a more pronounced and rapid current response compared to that of the pure NF electrode. The inset of Figure 6c shows that the average current response toward 350 μM glucose addition at the Cu NPs/NF electrode was approximately 2.49 mA cm^−2^, corresponding to a 2.9-fold enhancement relative to that of the pure NF electrode.

Furthermore, the real-time amperometric response of the Cu NPs/NF electrode toward incremental glucose additions was investigated. As presented in Figure 6d, the Cu NPs/NF electrode exhibited a rapid and pronounced stepwise increase in current response upon each addition of glucose, outperforming the pure NF electrode across the entire concentration range. The corresponding calibration curves were constructed by plotting the steady-state current density against glucose concentration (Figure 6e). The two-segment linear relationships of the Cu NPs/NF electrode were determined as Δ*j* (mA cm^−2^) = 0.007 *C* (μM)–0.023 (R^2^ = 0.991) in the range of 0.25–980.4 μM and Δ*j* (mA cm^−2^) = 0.0037 *C* (μM) + 1.65 (R^2^ = 0.965) in the range of 980.4–3310.5 μM, while the linear relationships of pure NF electrode were determined as Δ*j* (mA cm^−2^) = 0.003 *C* (μM)–0.026 (R^2^ = 0.994) and Δ*j* (mA cm^−2^) = 0.0012 *C* (μM) + 1.32 (R^2^ = 0.960) over the same concentration windows. The sensitivity of the Cu NPs/NF electrode was calculated to be 7000 and 3700 μA mM^−1^ cm^−2^ in the two linear ranges, respectively. The reduced sensitivity in the second segment (980.4–3310.5 μM) likely arises from the gradual saturation of surface-active sites and the accumulation of adsorbed reaction intermediates at higher glucose concentrations, which partially hinder further glucose access to the electrode surface. Notably, these sensitivities are substantially higher than those of the pure NF electrode (3000 and 1200 μA mM^−1^ cm^−2^). This pronounced improvement in amperometric response is attributable to the high loading of electrocatalytically active Cu nanoparticles uniformly dispersed on the NF scaffold, which dramatically increases the density of accessible active sites and facilitates more efficient charge transfer during glucose oxidation. The increased accessible active sites and the highly conductive framework synergistically facilitate efficient charge transfer during glucose electro-oxidation, thereby enabling the rapid, sensitive, and stable amperometric response. In addition, the Cu NPs/NF electrode achieved a remarkably rapid response time of approximately 3 s (Figure 6f), further indicating its competitive analytical performance and suitability for real-time glucose detection. The Cu NPs/NF electrode delivers competitive overall sensing performance, including a high sensitivity, a wide linear range, and a low detection limit, as summarized in Table 1, where it compares favorably with representative Cu-based non-enzymatic glucose sensors. The enhanced sensing performance arises from two synergistic factors: (i) the intimate Cu^0^/Cu^2+^ mixed-valence interface facilitates rapid electron transport between the active Cu^3+^ sites and the conductive substrate, and (ii) the macroporous NF framework provides a continuous conductive network for electron collection while enabling unimpeded electrolyte and glucose diffusion.

#### 3.2.4. Selectivity, Reproducibility, Storage Stability, and Real Sample Measurements

**Figure 7:**
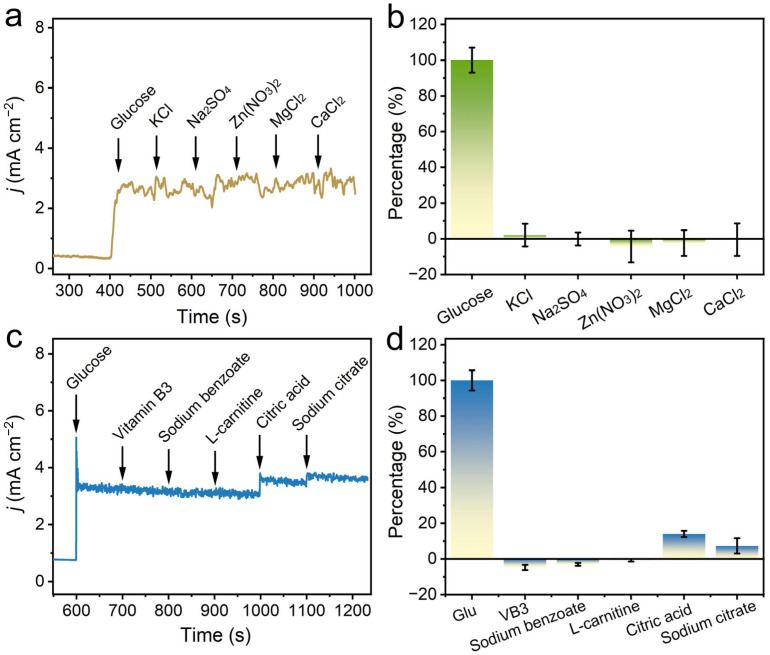
(**a**) Amperometric i–t curve of the Cu NPs/NF electrode recorded in 0.1 M NaOH with the addition of glucose and various inorganic salt interferents. (**b**) Corresponding column chart of the amperometric response. (**c**) Amperometric i–t curve of the Cu NPs/NF electrode recorded in 0.1 M NaOH with the addition of glucose and various small-molecule organic interferents. (**d**) Corresponding column chart of the amperometric response. Data are presented as mean ± SD (*n* = 3 independent electrodes).

Good selectivity, reproducibility, and storage stability are essential for the practical application of electrochemical glucose sensors. To evaluate the anti-interference capability of the Cu NPs/NF electrode, several common interfering species were selected. Amperometric responses were recorded at a constant applied potential of 0.6 V in 0.1 M NaOH upon successive additions of 350 μM glucose and inorganic salt interfering species, including KCl, Na_2_SO_4_, Zn(NO_3_)_2_, MgCl_2_, and CaCl_2_. As shown in Figure 7a,b, the Cu NPs/NF electrode exhibited a pronounced current response upon glucose injection, whereas the addition of the aforementioned interfering species generated negligible current responses, confirming the excellent selectivity toward glucose oxidation under the tested conditions. In addition, guided by the ingredient lists of the actual beverage samples, we expanded the selectivity study to include a series of small-molecule organic interferents. As shown in Figure 7c,d, the corresponding amperometric i-t curve shows that the Cu NPs/NF electrode generated a pronounced current response only upon glucose addition. It should be noted that citric acid and sodium citrate produced an amperometric response approximately 7~13% of that of glucose under the same detection conditions. This weak signal likely originates from the slow oxidation of citrate ions by the electrogenerated Cu(III) species at the electrode surface, as the multiple hydroxyl and carboxyl groups in citrate can interact with the active sites. However, the oxidation kinetics of citrate are substantially slower than those of glucose, resulting in only a marginal current.

**Figure 8:**
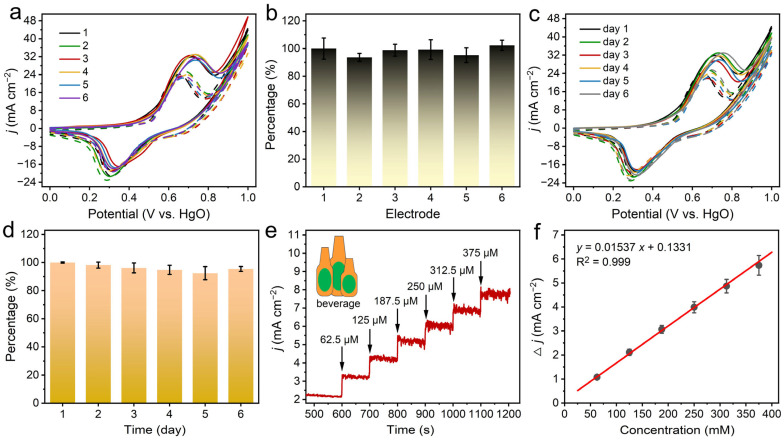
(**a**) CV curves of six dependently prepared Cu NPs/NF electrodes in 0.1 M NaOH with (solid line) and without (dash line) 1 mM glucose and (**b**) the corresponding column chart of the amperometric response. (**c**) CV curves of Cu NPs/NF electrodes stored for 1–7 days in 0.1 M NaOH with (solid line) and without (dash line) 1 mM glucose and (**d**) the corresponding column chart of the amperometric response. (**e**) Amperometric i–t curve of the Cu NPs/NF electrode recorded in 50-fold diluted beverage sample upon successive additions of glucose at 0.6 V and (**f**) the corresponding calibration curve of the response current density versus glucose concentrations. Data are presented as mean ± SD (*n* = 3 independent electrodes).

The reproducibility and storage stability of the Cu NPs/NF electrode were further evaluated through CV measurements. As depicted in Figure 8a,b, the CV curves of Cu NPs/NF electrodes prepared from different synthesis batches display consistent shapes and minimal variation in both the position and magnitude of the redox peaks, indicating highly reproducible electrode behavior. Figure 8c,d illustrate that the current response retention rates across electrodes stored for 1–7 days were consistently close to 100%. Furthermore, the redox characteristics, including peak potentials, current intensities, and overall voltametric profiles, exhibit remarkable consistency, demonstrating outstanding electrochemical reproducibility and short-term storage stability. These results demonstrate the exceptional reproducibility and storage stability of Cu NPs/NF electrode, which are essential for reliable sensing applications.

The practical applicability and reliability of the Cu NPs/NF electrode for glucose detection were further evaluated by performing standard addition and recovery experiments in commercial beverage samples. Prior to analysis, commercially obtained beverage samples were diluted 50-fold using 0.1 M NaOH solution to adjust the pH to the optimal alkaline sensing environment and to mitigate potential matrix effects. Amperometric responses were recorded at a constant applied potential of 0.6 V upon successive injections of known glucose concentrations to assess the accuracy of the method. As shown in Figure 8e,f, the Cu NPs/NF electrode exhibited a rapid and well-defined current increment upon each addition of glucose. The corresponding linear relationship was calculated as Δ*j* (mA cm^−2^) = 0.01537 *C* (μM)–0.1331 (R^2^ = 0.999). The standard calibration curve for the UV-Vis spectrophotometric method was established by plotting the absorbance at 510 nm versus glucose concentration (Figure S4). The linear regression equation was Abs = 0.694 *C* (mM) + 0.033, with R^2^ = 0.999 over the range of 0.055–2.22 mM. As summarized in Table 2, the original glucose concentration of the glucose oral rehydration solution determined by the electrochemical method was 6626.4 μM, in good agreement with the values obtained by the UV–Vis reference method (6396.1 μM), with relative deviations below 3.6%. Furthermore, standard addition recovery experiments determined from the amperometric response increments yielded recoveries ranging from 99.67% to 102.95%. The high recovery values confirm the practical viability of the Cu NPs/NF electrode for glucose detection in complex matrices and underscore its considerable potential for real-world analytical applications. However, the detection of real samples using the Cu NPs/NF electrode was limited to strongly alkaline conditions, diluted samples, and controlled laboratory settings. To address these limitations, future work will focus on evaluating long-term operational stability, investigating electrode fouling mechanisms, validating the sensor’s performance in more complex matrices, and integrating the sensor with a compact potentiostat and signal readout system to broaden its practical applicability.

## 4. Conclusions

In summary, a binder-free, self-supporting Cu NPs/NF electrode was successfully fabricated via a facile one-step hydrothermal method for non-enzymatic electrochemical glucose sensing. The in situ growth strategy effectively circumvented the limitations associated with conventional powder-coating techniques, such as buried active site and sluggish mass transport induced by polymetric binders. Comprehensive characterization using SEM, XRD, and XPS confirmed the successful formation of Cu NPs and their uniform dispersion across the three-dimensional NF scaffold. The highly conductive Cu NPs with high electrocatalytic activity enlarged the electrochemically active surface area and enhanced the electron-transfer kinetics during glucose oxidation. The as-prepared Cu NPs/NF electrode exhibited good electrocatalytic activity toward glucose oxidation in alkaline media. Under the optimized synthesis conditions, amperometric measurements demonstrated that the Cu NPs/NF-based sensor possesses a wide linear detection range of 0.25–3310.5 μM, a high sensitivity of 7000 μA mM^−1^ cm^−2^ in the low concentration region, a low detection limit of 0.32 μM, and a rapid response time of approximately 3 s. Furthermore, the electrode exhibited favorable selectivity, reproducibility, and storage stability. The practical viability of the proposed sensor was validated through standard addition and recovery experiments in real beverage samples, and the satisfactory recovery values confirmed its reliable analytical performance in complex matrices. This work not only provides a high-performance sensing platform suitable for food quality monitoring, but also highlights a cost-effective and scalable fabrication strategy for advanced binder-free electrocatalytic electrodes. Furthermore, beyond glucose detection, the facile and efficient synthesis process can be readily extended to the detection of other electroactive food-quality markers, offering considerable potential for the development of versatile multi-analyte sensing platforms.

## Figures and Tables

**Table 1:** Comparison of the sensing performance of the Cu NPs/NF electrode with that of other reported Cu-based non-enzymatic glucose sensors.

Sensor Material	Sensitivity (μA·mM^−1^·cm^2^)	Linear Range (mM)	LOD (μM)	Synthesis Method	Year	Refs.
Cu NPs/NF	7000; 3700	0.00025–3.31	0.32	Hydrothermal method	2026	This work
Cu foam/SPCE	2570; 1810	0.002–5	0.98	Electrodeposition assisted with hydrogen evolution	2014	[18]
Cu/Fe_3_O_4_/NF	12,850	0–1	0.71	Solvothermal method & reductive calcination	2025	[19]
Cu_2-x_Se/CP	5391; 3004	0.005–0.76	1.2	Electrodeposition method & chemical vapor deposition.	2023	[20]
CuNi foam	518	0.01–1	19.1	Electrodeposition	2025	[31]
Cu-NPG/SPE	659.9	0.001–13	0.13	Electrodeposition	2021	[32]
Cu_x_O-LIG	\	0–4	84	Laser direct writing & flash light irradiation	2026	[33]
TiO_2_/CuO	4559	0.05–6	50	Anodization & hydrothermal & annealing approach	2026	[34]
Cu-NGr	4846.94	0.00001–0.1	0.01	Chemical reduction	2017	[35]
Cu@Ni	420	0.2–12.2	62.5	Electrodeposition	2024	[36]
Cu_x_O/NPC@Co_3_O_4_/NPC-10-7	3314.29	0.0005–23.332	0.24	Solvothermal method & high temperature pyrolysis	2024	[37]

**Table 2:** The electrochemical quantification of glucose in beverage samples.

Sample	Added (μM)	Found (μM)	Recovery (%)	Glucose Found by UV–Vis (μM)	Recovery of UV-Vis (%)	RD (%)
Control	\	6626.4	\	6396.1	\	3.6%
1	75	74.75	99.67 ± 6.78	73.56	101.96 ± 5.12	
2	150	154.43	102.95 ± 7.13	170.53	87.96 ± 3.29	
3	225	228.48	101.55 ± 6.12	255.98	87.89 ± 5.97	

## Data Availability

The original contributions presented in this study are included in the article/Supplementary Material. Further inquiries can be directed to the corresponding authors.

## Supplementary Materials

The following supporting information can be downloaded at: https://www.mdpi.com/article/10.3390/foods15111993/s1, Figure S1: (a) The SEM image of Cu NPs/NF and (b) the corresponding particle size distribution of Cu NPs; Figure S2: The relative proportions of Cu^2+^ and Cu^+^/Cu^0^ species in the Cu NPs/NF; Figure S3: Schematic illustration of glucose electro-oxidation on Cu NPs/NF electrode; Figure S4: The standard calibration curve for the glucose detection by UV-Vis spectrophotometric method.

## Abbreviations

The following abbreviations are used in this manuscript:

NF	Ni foam
Cu NPs	Cu nanoparticles
ECSA	Electrochemically active surface area
CV	Cyclic voltammetry
EIS	Electrochemical impedance spectroscopy

## References

[1] Parker J. (2020). Glucose metabolism, energy production and regulation of cellular and whole-body metabolism. J. Australas. Coll. Nutr. Environ. Med..

[2] Starkey D.E., Wang Z., Brunt K., Dreyfuss L., Haselberger P.A., Holroyd S.E., Janakiraman K., Kasturi P., Konings E.J.M., Labbe D. (2022). The Challenge of Measuring Sweet Taste in Food Ingredients and Products for Regulatory Compliance: A Scientific Opinion. J. AOAC Int..

[3] Cheng S., Zhang M., Cong X., Li J., Shi Q., Min J.Z. (2026). Sweeteners in Diabetes and Blood Glucose Management: Advances, Challenges, and Trends in the Food Industry. Food Rev. Int..

[4] Akyüz E., Başkan K.S., Tütem E., Apak R. (2021). High performance liquid chromatographic method with post-column detection for quantification of reducing sugars in foods. J. Chromatogr. A.

[5] Lee T., Park J., Oh S.H., Cheong D.Y., Roh S., You J.H., Hong Y., Lee G. (2024). Glucose Oxidase Activity Colorimetric Assay Using Redox-Sensitive Electrochromic Nanoparticle-Functionalized Paper Sensors. ACS Omega.

[6] Dominguez R.B., Orozco M.A., Chávez G., Márquez-Lucero A. (2017). The Evaluation of a Low-Cost Colorimeter for Glucose Detection in Salivary Samples. Sensors.

[7] Fan S., Wang J., Wen X., Feng Y., Shao F., Xu L., Liu S., Liu W. (2026). A label-free fluorescence polarization technology for the detection of food additives. Spectrochim. Acta Part A Mol. Biomol. Spectrosc..

[8] Jiang S., Li Q., Wu G., Mu X., Wang X., Wang Y., Wu Y., Wu J., Li Y. (2024). Advances in Label-Free Glucose Detection Using Self-Assembled Nanoparticles and Surface-Enhanced Raman Spectroscopy. Anal. Chem..

[9] Shakya A.K., Ramola A., Balal N., Bergman A. (2026). Evolution of Photonic Crystal Fiber-Based Smart Sensors with Emphasis on Biomedical Applications: A Review. IEEE Sens. J..

[10] Zhang C., Lai Q., Chen W., Zhang Y., Mo L., Liu Z. (2023). Three-Dimensional Electrochemical Sensors for Food Safety Applications. Biosensors.

[11] Manikandan V.S., Adhikari B., Chen A. (2018). Nanomaterial based electrochemical sensors for the safety and quality control of food and beverages. Analyst.

[12] Wang G., He X., Wang L., Gu A., Huang Y., Fang B., Geng B., Zhang X. (2013). Non-enzymatic electrochemical sensing of glucose. Microchim. Acta.

[13] Wang H., Zhu W., Xu T., Zhang Y., Tian Y., Liu X., Wang J., Ma M. (2022). An integrated nanoflower-like MoS_2_@CuCo2O4 heterostructure for boosting electrochemical glucose sensing in beverage. Food Chem..

[14] Manna A.K., Guha P., Srivastava S.K., Varma S. (2024). Non-enzymatic glucose sensors based on electrodeposited CuxO–ZnO composite nanostructures. J. Mater. Sci. Mater. Electron..

[15] Shen N., Xu H., Zhao W., Zhao Y., Zhang X. (2019). Highly Responsive and Ultrasensitive Non-Enzymatic Electrochemical Glucose Sensor Based on Au Foam. Sensors.

[16] Wu P., Fan J., Tai Y., He X., Zheng D., Yao Y., Sun S., Ying B., Luo Y., Hu W. (2024). Ag@TiO_2_ nanoribbon array: A high-performance sensor for electrochemical non-enzymatic glucose detection in beverage sample. Food Chem..

[17] Aun T.T., Salleh N.M., Ali U.F.M., Manan N.S.A. (2023). Non-Enzymatic Glucose Sensors Involving Copper: An Electrochemical Perspective. Crit. Rev. Anal. Chem..

[18] Niu X., Li Y., Tang J., Hu Y., Zhao H., Lan M. (2014). Electrochemical sensing interfaces with tunable porosity for nonenzymatic glucose detection: A Cu foam case. Biosens. Bioelectron..

[19] Xu J., Cai H., Yu K., Hou J., Li Z., Zeng X., He H., Zhang X., Su D., Yang S. (2025). Self-Supported Cu/Fe_3_O_4_ Hierarchical Nanosheets on Ni Foam for High-Efficiency Non-Enzymatic Glucose Sensing. Nanomaterials.

[20] Wang C., Yang X., Wang T., Zhu G., Luo Z., Jiao P., Yu D., Yu H. (2023). Copper Selenide Micro/Nanocrystals on Carbon Paper via Chemical Vapor Deposition Selenylation as Electrodes for Non-enzymatic Glucose Sensing. ACS Appl. Nano Mater..

[21] Yang B., Liu H., Gao J., Xu J., Pang L. (2025). Fabrication of three dimensional NiO nanosheet arrays on carbon cloth as self-supporting non-enzyme glucose electrochemical biosensor. J. Alloys Compd..

[22] Wang C., Han B., Li J., Gao Q., Xia K., Zhou C. (2021). Direct epitaxial growth of nickel phosphide nanosheets on nickel foam as self-support electrode for efficient non-enzymatic glucose sensing. Nanotechnology.

[23] Akbarzadeh R., Dehghani H. (2014). A novel thermal reduction method towards the synthesis and growth of two unlike morphologies of nickel nanostructures. Dalton Trans..

[24] Qiu Y., Tan G., Xu P., Luo Q., Lin X., Huang W., Li J. (2015). Preparation of Cu(OH)_2_ and ZnO nanoarrays on surface of metal substrates by a simple method and application as ammonia sensors. Appl. Surf. Sci..

[25] Zhu Y., Kong X., Li X., Ding G., Zhu Y., Li Y.-W. (2014). Cu Nanoparticles Inlaid Mesoporous Al_2_O_3_ As a High-Performance Bifunctional Catalyst for Ethanol Synthesis via Dimethyl Oxalate Hydrogenation. ACS Catal..

[26] Jiang Y., Lv C., Lu B., Song Y., Liu T., Zhang X., Gao D., Ye K., Wang G. (2025). Ag Stabilized Cu^+^/Cu^0^ Interface Catalysts for Enhanced CO_2_ Electroreduction to C2+ Products at Ampere Level Current Density. ACS Nano.

[27] Shao H.-X., Xu H., Xu M.-M., Zheng H.-Z., Zhou M., Ren F.-Y., Wu J.-J., Zhang Y., Zhao J., Zhao B. (2026). Effective CO_2_ Photoreduction Synergistically Boosted by Built-In Electronic Field and Coplanar Covalent Triazine Frameworks. J. Am. Chem. Soc..

[28] Mei J., Guo R., Wang D., Shang J., Zhao J., Bai J., Yao Q., Dou S. (2026). Support-Intensified Ir—P/O—Mo Cooperative Linkages for Robust Acidic Water Dissociation. Adv. Sci..

[29] Luo S., Yang M., Li J., Wu Y. (2023). One-step potentiostatic electrodeposition of NiS–NiS_2_ on sludge-based biochar and its application for a non-enzymatic glucose sensor. RSC Adv..

[30] Zhu L., Ma J., Jin D., Zhang Y., Wu S., Xu A., Gu Y., An Y., Miao Y. (2023). Flower-like WSe_2_ used as bio-matrix in ultrasensitive label-free electrochemical immunosensor for human immunoglobulin G determination. Anal. Sci..

[31] Kim D., Choi S., Lim H.-R. (2026). Strategically managing all electrodes in screen-printed platforms to enable high performance non-enzymatic 3D CuNi foam-based glucose sensors. Microchem. J..

[32] Hernández-Saravia L.P., Martinez T., Llanos J., Bertotti M. (2021). A Cu-NPG/SPE sensor for non-enzymatic and non-invasive electrochemical glucose detection. Microchem. J..

[33] Lee G.H., Son S.E., Kim J.M., Lee S., Koo Y., Lee M., Shin J.Y., Jeong D.W., Lee G., Cho D. (2026). A copper oxide-modified laser-induced graphene electrode for the non-enzymatic and simultaneous detection of glucose and creatinine. Appl. Surf. Sci..

[34] Fahad N.K., Ameer Z.S.A.-A., Shakir Z.H., Abdaldeen Z.K., Rahmah M.I. (2026). Impact of time anodization synthesis of TiO_2_/CuO/Cu heterojunction nanostructures for high-performance glucose biosensors. J. Mater. Sci. Mater. Electron..

[35] Shabnam L., Faisal S.N., Roy A.K., Haque E., Minett A.I., Gomes V.G. (2017). Doped graphene/Cu nanocomposite: A high sensitivity non-enzymatic glucose sensor for food. Food Chem..

[36] Ghosh R., Li X., Yates M.Z. (2024). Nonenzymatic Glucose Sensor Using Bimetallic Catalysts. ACS Appl. Mater. Interfaces.

[37] Cao W., Guo T., Wang J., Ding Y., Fan B., Liu D. (2024). Hierarchical N-doped porous carbon scaffold Cu/Co-oxide with enhanced electrochemical sensing properties for the detection of glucose in beverages and ascorbic acid in vitamin C tablets. Food Chem..

