# Supplementary material for: Facile In Situ Synthesis of Self-Supporting Cu Nanoparticles/Nickel Foam Electrode for Sensitive Non-Enzymatic Electrochemical Glucose Sensing in Beverages
Source: Foods. 2026 Jun 3;15(11):1993. doi: 10.3390/foods15111993 (PMC13256642; doi:10.3390/foods15111993)
Supplement: Supplementary file 1 [file foods-15-01993-s001.zip › foods-4313583-supplementary.pdf]

Supplementary Information

Facile In-Situ Synthesis of Self-Supporting Cu Nanoparticles/Nickel Foam for Sensitive Nonenzymatic Electrochemical Glucose Sensing in Beverage.

Yanlin Wu [1], Xintian Ma [1], Yiyue Ma [1,*] and Jianlong Wang [1,*]

[a] College of Food Science and Engineering, Northwest A&F University, Yangling 712100, Shaanxi, China

* Corresponding author. E-mail: wanglong79@nwsuaf.edu.cn (J. Wang)

* Corresponding author. E-mail: yiyuema@163.com (Y. Ma)

Numbers of pages: 5

Numbers of figures: 4

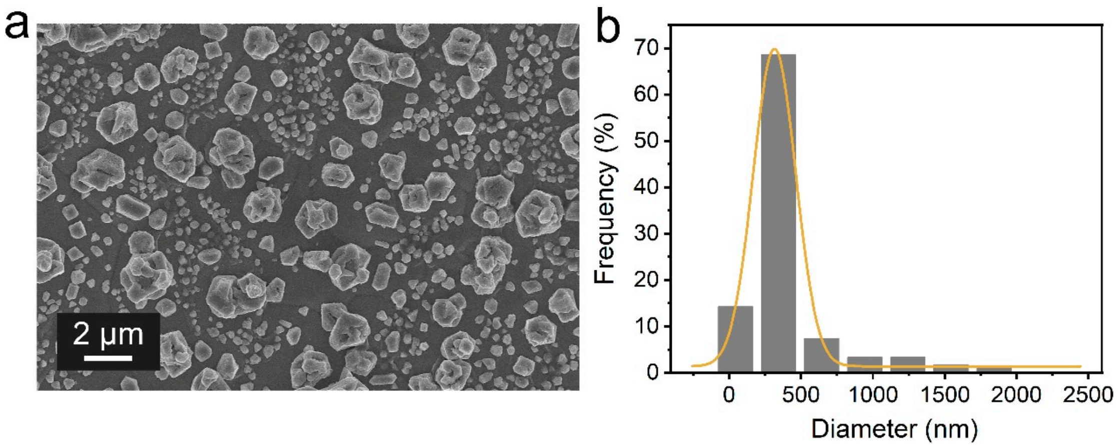

Figure S1. (a) The SEM image of Cu NPs/NF and (b) the corresponding particle size distribution of Cu NPs.

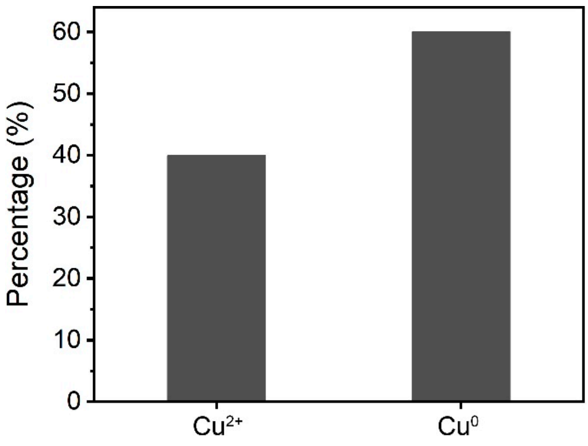

Figure S2. The relative proportions of Cu^{2+} and Cu^{+}/Cu^{0} species in the Cu NPs/NF.

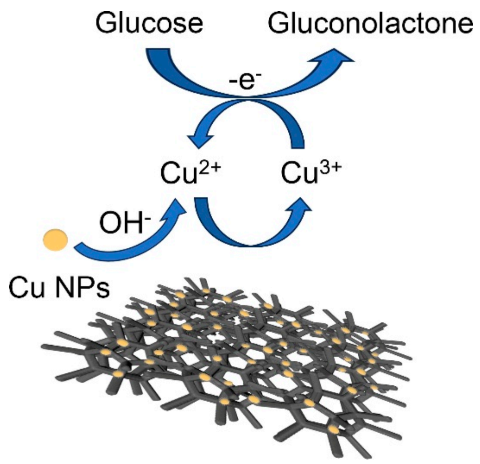

Figure S3. Schematic illustration of glucose electro-oxidation on Cu NPs/NF electrode.

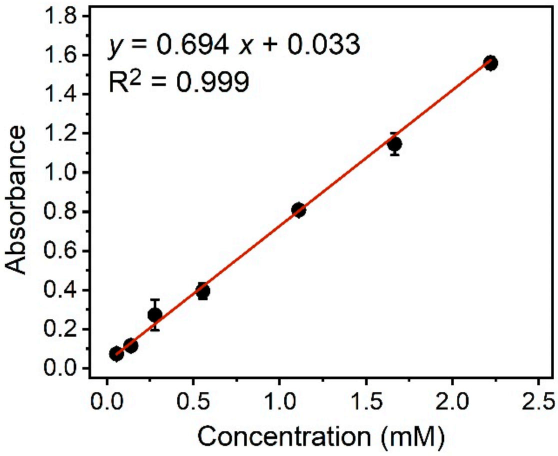

Figure S4. The standard calibration curve for the glucose detection by UV-Vis spectrophotometric method.
